# A Novel Denture Labelling Technique for Human Identification

**DOI:** 10.7759/cureus.32740

**Published:** 2022-12-20

**Authors:** Shreya Colvenkar, Suman Pathipaka, SRN Venkata Harish V, Anish Polasi, Kasukurthi Vijay Kumar

**Affiliations:** 1 Department of Prosthodontics, MN Raju (MNR) Dental College and Hospital, Sangareddy, IND; 2 Department of Prosthodontics, Ganni Subba Lakshmi (GSL) Dental College & Hospital, Rajamahendravaram, IND; 3 Department of Prosthodontics, Drs Sudha & Nageswara Rao Siddhartha Institute of Dental Sciences, Vijayawada, IND

**Keywords:** labelling, photograph, aadhar number, metal, marker, denture

## Abstract

The importance of denture labelling has been acknowledged worldwide by different forensic organizations. Denture labelling is important to identify victims of crimes, mass disasters, accidents, patients with memory loss as well as misplaced dentures in old-age homes. This article describes a simple technique of incorporating a metallic marker in the denture. The metallic marker is inscribed with the patient's photograph and Aadhaar number (a unique identification number issued to Indian residents by the Unique Identification Authority of India). The photograph would help in day-to-day identification, e.g., in old-age homes and large hospital setups. The Aadhaar number would provide additional details of the individuals, thus ensuring quick identification. The proposed method is simple and cheap, with the marker easily readable and resistant to high temperature, thus satisfying the ideal requirements of a denture marker.

## Introduction

Human identification is essential for forensic and social scenarios, bringing a positive closure to the case [1]. Dental records like missing teeth, restorations, implants, radiographs, surgical stents, and braces provide a vital clue during forensic investigation. An edentulous patient with missing teeth fails to provide a good record during investigation. Hence, labelling of dentures is important to know details of the patient.

Denture marking avoids mixing and misplacing of dentures in geriatric facilities and hospital setups [2]. It also helps to identify victims of natural disasters like earthquakes, tsunami, floods as well as aviation disasters. Lost dentures that are labelled can be returned to the owner in the case of accidents, unconsciousness or amnesia.

The literature mentions various methods for denture labelling. They are briefly classified into surface marking and inclusion methods [3-13]. Surface marking techniques are not permanent and store very little data [3,4]. In inclusion techniques, different types of metallic as well as non-metallic markers are added as a marker [5]. Dentists should brief the patients about different types of denture markers, their advantages and disadvantages.

This article describes a simple technique of incorporating a metallic marker with a patient's photograph and Aadhaar number (a unique identification number issued to Indian residents by the Unique Identification Authority of India) in the patient's denture. The photographic marker would help in quick identification as it is easily readable by a lay person. It would take time to gather Aadhaar data of a patient as only specialized government agencies would have access to the details; hence, incorporating the photograph is important. The additional information about the patient would be found out with the help of the Aadhaar number during a forensic investigation.

## Technical report

**Figure 1:**
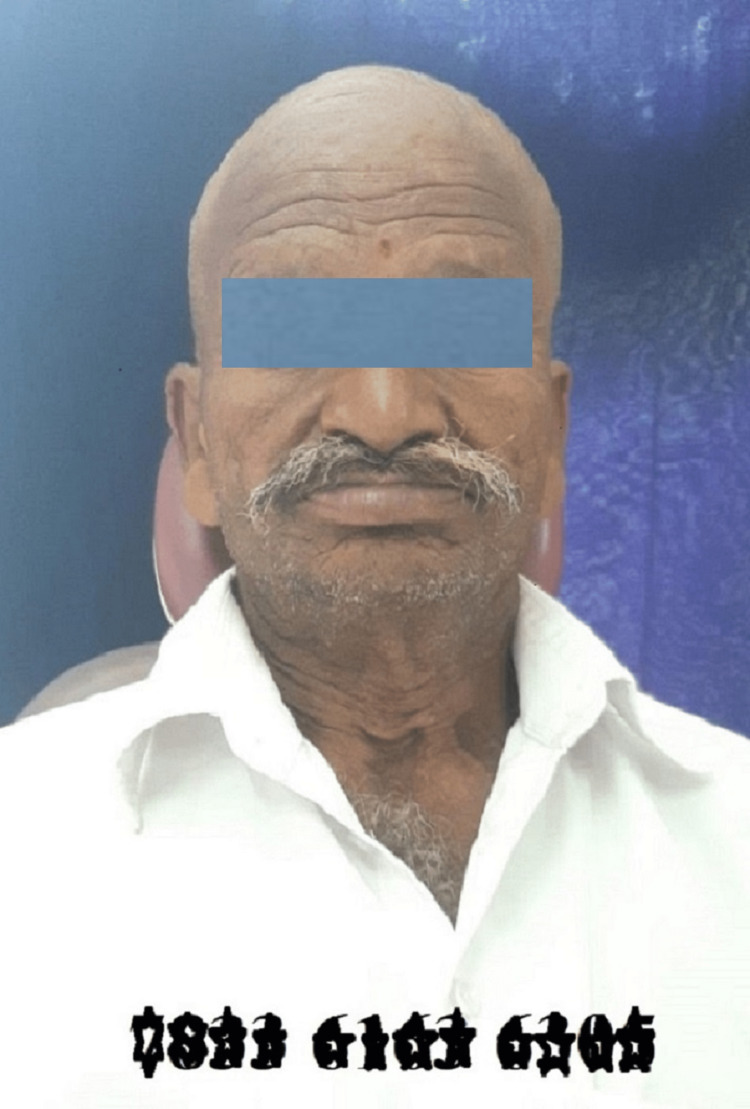
Patient's photograph with the Aadhaar number

First, a patient's photograph and Aadhaar number are taken and they are collaged together using an editing software (Figure 1).

**Figure 2:**
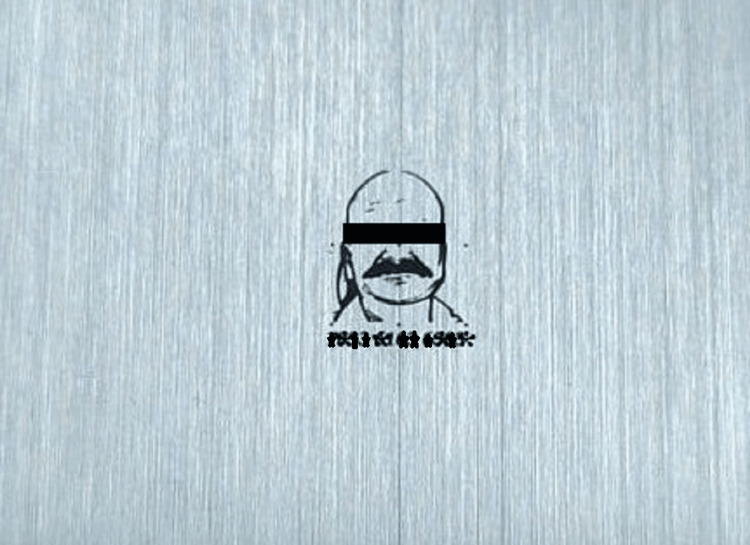
Patient's photograph and Aadhaar number laser marked

Then, the patient photograph as well as the Aadhaar number are laser marked on a stainless steel sheet measuring 8 x 8 x 0.2 mm in thickness (Figure 2).

**Figure 3:**
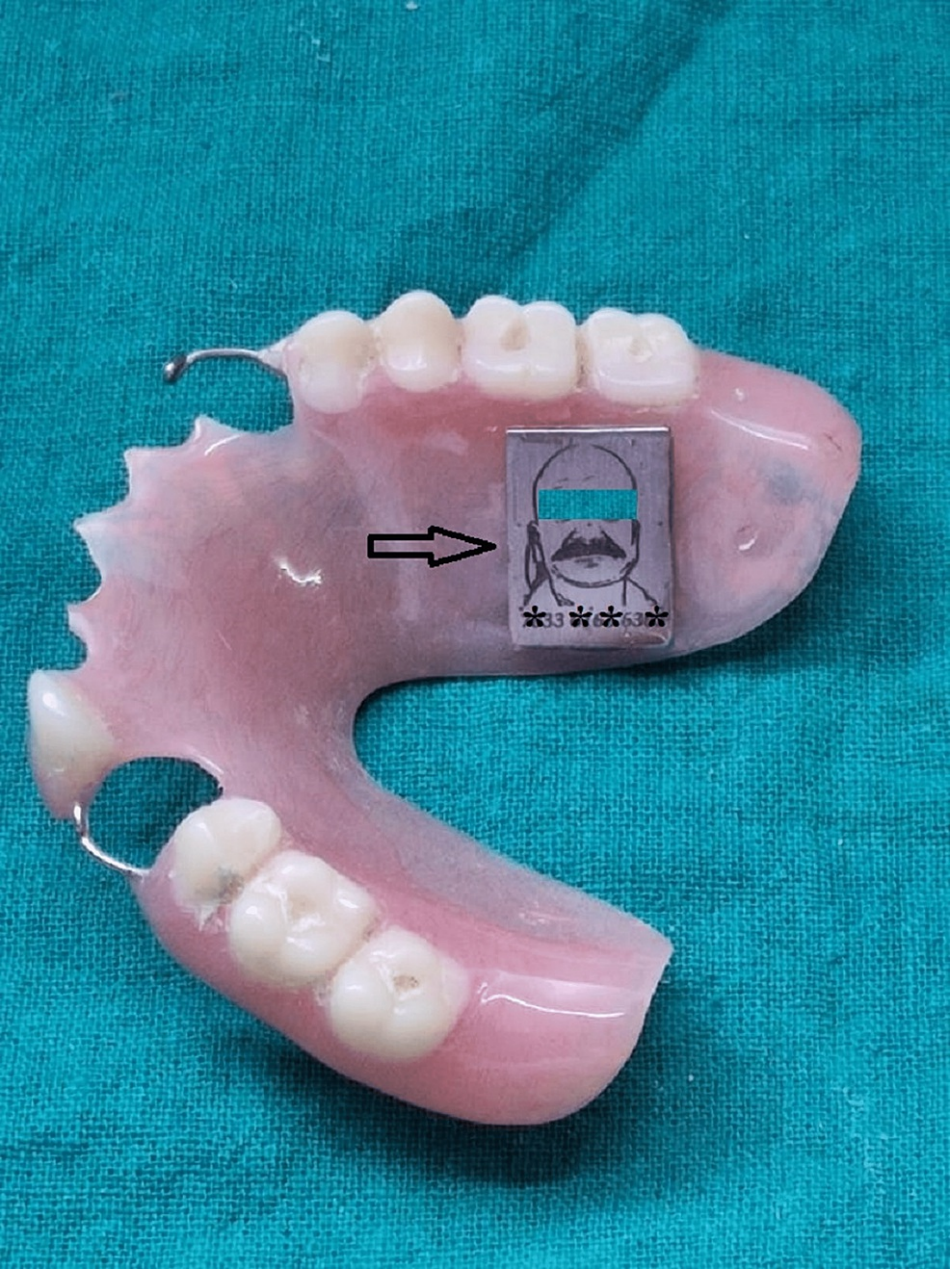
The marker inserted in the denture

Next in the method, the dentures are processed using a conventional technique according to the manufacturer's instructions. The maxillary denture is disinfected, cleaned and dried. Using a carbide bur (Zhangjiagang Saimeng Tools Co., Ltd., Jiangsu, China), a depression wider than the marker is made on the palatal surface of the denture. The marker is placed in the recess and the surface is primed with the metal primer. The recess is covered with clear, autopolymerized acrylic resin (Rapid Repair; Dentsply Int'l, York, PA). The denture (Confident Dental Equipments Private Limited, Bangalore, India) is processed with warm water (1008F, 20 psi) for 15 to 20 minutes in a pressure pot (Confident Dental Equipments Private Limited, Bangalore, India). The denture is finally polished (Figure 3).

## Discussion

Denture labelling assists in finding owners of misplaced or lost dentures. It also helps to provide a vital clue of the denture wearer, either deceased or living. Denture marking has been recommended by various forensic odontologists and forensic organizations worldwide. Numerous criteria have been put forth by the American Dental Association for denture marking. The markers should be specific, simple, fire and solvent resistant, aesthetic and should not weaken the denture.

Various techniques have been mentioned in the literature for denture marking that include surface marking and inclusion technique. In surface marking, patients' initials or details are scribed with a fiber tip pen, spirit-based pen, or invisible ink pen on the denture surface [3]. Few authors also report of engraving the details on the denture surface [4]. Surface marking techniques are simple, cheap and quick, but markers are not fire resistant and store more information. In surface inclusion techniques, various markers like lenticular card [6], microchip [7], micro-SD card [8], stainless steel band [9] and metal barcodes [10] are incorporated in the denture, but none of them satisfy all the requirements of denture markers in terms of cost, storage or resistance to high temperature.

The method described here, to include a metallic marker with the photograph and Aadhaar number, surpassed other denture marking methods in various ways. A photograph, being easily recognizable, plays a crucial role in day-to-day identification in geriatric facilities, old-age homes and large hospital setups [11]. It also helps in preventing the misplacement of the dentures among multilingual and illiterate denture wearers in geriatric facilities. Also, additional patient information, such as name, age, mobile number, gender, photograph, enrollment number and address of the individual, can be retrieved from a patient's Aadhaar number. Aadhaar is a 12-digit number provided by the Government of India and is linked to individual biometrics providing robust and unique identity to each and every resident of India. The Aadhaar number has been used on surgical plates and dentures as seen in previous studies, and using the Aadhaar number would provide rapid access to the patient's information [13,14].

As the marker is metallic, it is durable and can withstand high temperatures. Metal markers are ideal for postmortem identification especially during aviation disasters [9]. A study conducted by Anehosur et al. concluded that photographic markers and metal matrix bands were easy to incorporate, but fire resistance of photographic markers and barcodes was less compared to the metal band [12]. The authors had used paper-based photographic markers. Colvenkar and Ravindra mentioned the use of a stainless steel metallic marker with a barcode in the denture [10]. In the present technique, considering the advantages of a metallic marker over a paper-based marker, patients' photograph with the Aadhaar number was printed on a stainless steel sheet. An additional advantage is that laser marking allows the size of the marker to be adjusted to the smallest possible size using computer software.

The cost of printing a photograph with the Aadhaar number on a metal sheet was only 6 USD. The marker can be inserted in the lingual flange of the mandibular and buccal flange of the maxillary denture without compromising the aesthetics and function. The metal surface is primed with a primer before the addition of autopolymerized acrylic resin. Priming increases the bond strength between stainless steel and autopolymerized acrylic resin, and hence, there is no chance of accidental ingestion. The study conducted by Shimpo et al. concluded that the primed specimens significantly improved the bond strengths between the denture acrylic resin and stainless steel [15]. Future studies need to be carried out to know the strength of dentures with incorporated markers and their resistance to everyday cleansing and disinfection methods.

The method of incorporating a photograph with the Aadhaar number can be used to label new dentures and old dentures of patients that have not been previously labelled. There is no need for special instruments, and dentists can easily carry out the technique in a dental setup with readily available materials.

## Conclusions

Here we have described a simple technique of incorporating a metallic marker with a patient's photograph and Aadhaar number in the patient’s denture. The photographic marker being easily readable can be helpful in the day-to-day identification of patients in old-age homes. Also, additional information about a patient can be provided by the patient's Aadhaar number. As the marker is metallic, it is durable and can withstand high temperatures.

## References

[1] Borrman HI, DiZinno JA, Wasén J, René N (1999). On denture marking. J Forensic Odontostomatol.

[2] Stenberg I, Borrman HI (1998). Dental condition and identification marking of dentures in homes for the elderly in Göteborg, Sweden. J Forensic Odontostomatol.

[3] Stevenson RB (1987). Marking dentures for identification. J Prosthet Dent.

[4] Stavrianos C, Petalotis N, Metska M, Stavrianou I, Papadopoulos C (2007). The value of identification marking on dentures. Balk J Stom.

[5] Ling BC (1998). Computer-printer denture microlabeling system. J Prosthet Dent.

[6] Colvenkar SS (2010). Lenticular card: a new method for denture identification. Indian J Dent Res.

[7] Millet C, Jeannin C (2004). Incorporation of microchips to facilitate denture identification by radio frequency tagging. J Prosthet Dent.

[8] Colvenkar SS, Gopal S (2014). Micro secure digital card: a novel method for denture identification. J Forensic Dent Sci.

[9] Stavrianos C, Stavrianou I, Kafas P (2008). Denture identification system based on Swedish guidelines: a forensic aspect. J Forensic Sci.

[10] Colvenkar S, Ravindra SV (2022). Denture marking for forensic identification using laser-marked stainless steel quick response (QR) code. Cureus.

[11] Colvenkar S, Alwala AM, Kunusoth R, Sampreethi S, Devera Shetty S (2022). A simple denture-marking technique for patients residing at old age homes. Cureus.

[12] Anehosur GV, Acharya AB, Nadiger RK (2010). Usefulness of patient photograph as a marker for identifying denture-wearers in India. Gerodontology.

[13] Priya PH, Jei JB, Murugesan K (2021). Role of unique identification number and barcode of Aadhaar in forensic odontology. Saudi J Oral Dent Res.

[14] Prakash R, Colvenkar S, Alwala AM, Katkuri S, Ahmed MS (2022). Aadhar number marking on surgical plate for forensic identification. Cureus.

[15] (2009). Shear bond strengths of denture acrylic resin to stainless steel. https://iadr.abstractarchives.com/abstract/2009miami-118141/shear-bond-strengths-of-denture-acrylic-resin-to-stainless-steel.

